# Feasibility of prevention of type 2 diabetes in low- and middle-income countries

**DOI:** 10.1007/s00125-023-06085-1

**Published:** 2024-02-15

**Authors:** Andre P. Kengne, Ambady Ramachandran

**Affiliations:** 1 Non-Communicable Diseases Research Unit, South African Medical Research Council, Cape Town, South Africa; 2 Department of Medicine, Faculty of Health Sciences, University of Cape Town, Cape Town, South Africa; 3 Department of Biological and Environmental Sciences, Faculty of Natural Sciences, Walter Sisulu University, Mthatha, South Africa; 4 Indian Diabetes Research Foundation, Chennai, India; 5 Dr. A. Ramachandran’s Diabetes Hospitals, Chennai, India

**Keywords:** Diabetes, Impaired fasting glucose, Impaired glucose tolerance, Low- and middle-income countries, Primary prevention, Review, Screening

## Abstract

Type 2 diabetes is a leading cause of global mortality and morbidity. Nearly 80% of individuals with diabetes live in low- and middle-income countries (LMICs), where nearly half of those with the condition remain undiagnosed. The majority of known cases have sub-optimal clinical outcomes. Moreover, large populations with impaired glucose tolerance and/or impaired fasting glucose contribute to the rapid increase in type 2 diabetes. Globally, priority should be given to limit the population with diabetes, especially in LMICs, alongside actions to optimise the care of people diagnosed with diabetes. Primary prevention studies in LMICs have generated evidence to show the efficacy and scalability of strategies to fully prevent or delay the development of diabetes in high-risk groups. However, these are mainly limited to certain countries in Asia, particularly China and India. The studies have indicated that prevention policies are effective in populations with a high risk of type 2 diabetes, and they also have long-term benefits, not only for the risk of type 2 diabetes but also for the risk of associated metabolic disorders, such as CVDs. For the effective conduct of national programmes, innovative mechanisms must be implemented, such as the use of information technology, joint efforts of multiple teams implementing similar programmes, and involvement of governmental and non-governmental partnerships. Continuous monitoring and long-term studies are required to assess the utility of these programmes. The effectiveness of such programmes in LMICs has not been proven over the longer term, except in China. Despite the available evidence, the feasibility of prevention strategies for type 2 diabetes in LMICs at population level remains an enigma. There remain challenges in the form of cultural, societal and economic constraints; insufficient infrastructure and healthcare capacity; and the non-fully elucidated natural history and determinants of type 2 diabetes in LMICs.

**Graphical Abstract:**

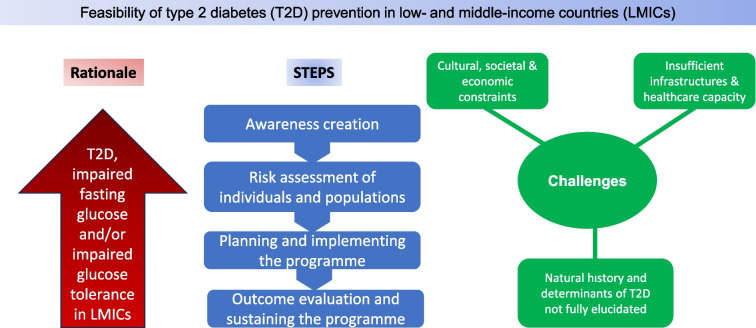

**Supplementary Information:**

The online version contains a slide of the figure for download available at 10.1007/s00125-023-06085-1.

## Introduction

Leading expert committees, including the IDF [1], the Non-Communicable Diseases Risk Factors Collaboration (NCD-RisC) [2] and the Global burden of disease (GBD) study group [3], have highlighted the worldwide increase in the population of people with diabetes mellitus, and its related health impact and expenditures [1–4]. The rising burden of diabetes is very high in the low- and middle-income countries (LMICs), where the condition is not optimally recognised, detected and treated [5]. According to the latest reports in the IDF Diabetes Atlas [1], about 81% of the 537 million adults aged 20–79 years with diabetes in 2021 were from LMICs, mainly constituting the countries of the Middle East, sub-Saharan Africa and the Indian subcontinent. Nearly half of those with diabetes remain undetected in these countries, which corresponds to 87.5% of undiagnosed diabetes worldwide [1]. It is also predicted that by 2045, the total number of individuals with diabetes will increase to 783 million, and that 85% of these will be residing in LMICs. The major increase in diabetes will occur due to conversion from prediabetes (i.e. impaired fasting glucose [IFG] and impaired glucose tolerance [IGT]). In 2021, an estimated 541 million adults had IGT (424.5 million from LMICs), while 319 million had IFG (254.4 million from LMICs) [1].

Global efforts to contain the burden of diabetes should prioritise actions in LMICs and should be two-pronged, including: (1) optimising the timely detection of those with undiagnosed diabetes and linking them to care to ensure adequate management, thus limiting the risk of long-term complications; (2) preventing further increase of the population with diabetes. A study covering 55 LMICs concluded that only 43.9% of people with diabetes were aware of their status, among whom only 4.5% reported meeting the need for all treatments recommended to them [6]. Studies hitherto have shown definite evidence that the onset and progression of type 2 diabetes can be postponed or prevented [4, 5, 7, 8]. However, the challenges in preventing the disorder in the real-world setting are huge; even in developed countries, practical problems exist [9]. Translating the findings of prevention programmes at population level are possible, but it is an uphill task.

**Fig. 1:**
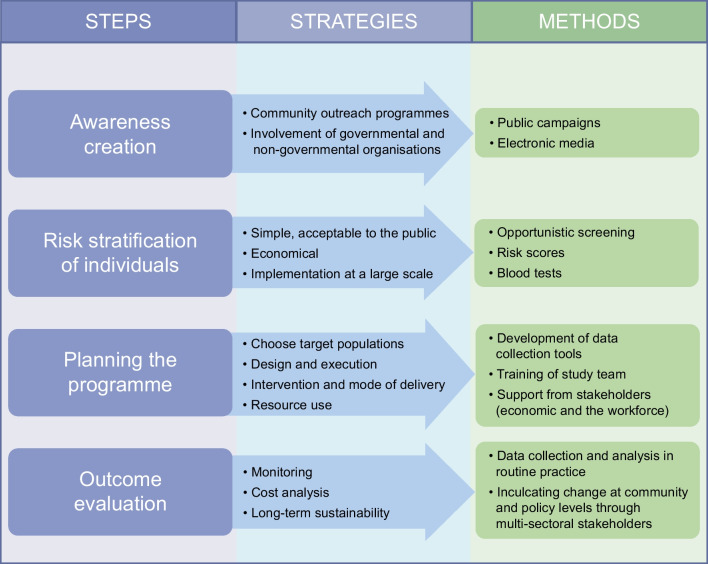
Steps in the implementation of effective type 2 diabetes prevention programmes: the way forward. Four steps are distinguished: (1) awareness creation; (2) risk stratification of individuals; (3) planning the programme; and (4) outcome evaluation. For each of the steps, corresponding strategies and methods for achieving them are outlined. This figure is available as a downloadable slide

A gene–environmental interaction is associated with the development of clinical diabetes. Environmental factors mainly change dietary pattern and sedentary behaviour, leading to obesity superimposed on genetic and epigenetic susceptibility [10]. Changes in environmental risk factors caused by socioeconomic transition produce adverse biological effects and lead to expression of diabetes. Therefore, prevention of type 2 diabetes must focus on the above interrelated factors. In this review we focus on the recent strategies used in the primary prevention of type 2 diabetes that are appropriate for LMICs. We also highlight the hurdles that exist in preventing type 2 diabetes in LMICs while discussing the steps in implementing effective prevention programmes (see Fig. 1).

## Burden and trends of diabetes and impaired fasting glucose and/or impaired glucose tolerance in LMICs

According to the IDF estimates, in 2021 the comparative age-standardised prevalence of diabetes among those aged 20–79 years was 9.8% overall, 8.4% in high-income countries (HICs), 10.5% in middle-income countries (MICs) and 6.7% in low-income countries (LICs) [1]. Of the estimated 537 million adults with diabetes in 2021, 104 million (19.4% of the total) were from HICs, 414 million (77.1%) were from MICs and 19 million (3.5%) were from LICs [1].

The GBD, IDF and NCD-RisC have consistently reported worldwide increases in diabetes burden in recent decades, and have also suggested likelihood of further increases in the next few decades. Among other factors, this is predicted to be driven by rapid urbanisation and globalisation (which create a favourable context for the uptake and augmentation of diabetes risk factors) and ageing of the population. Diabetes prevalence rates in 2014 were at least five times higher in LMICs, such as North Africa and the Middle East, when compared with some Western European countries [2, 3]. Studies from India have also shown the increasing burden of diabetes, not only in urban areas but also in the rural regions [11, 12].

The prevalence of diabetes and IGT and/or IFG is increasing rapidly in all countries, especially in LMICs where prevention and control tasks are highly challenging because of the poor socioeconomic status. Moreover, some populations, such as South Asian populations, have a distinctive genotype and phenotypical characteristics that affect the pathophysiology of diabetes, influencing the ability of lifestyle interventions to reverse diabetes. Moreover, abnormal metabolic characteristics, such as insulin resistance, occur earlier in life in this population, most commonly in the first two decades [13–15]. In addition, in early childhood, South Asian individuals are more insulin resistant than white populations [14, 15]. There is evidence to show that, in LMICs, such as Africa, malnutrition in utero and after birth leads to early insulin resistance and dysglycaemia in children born with low birthweight [16]. Sattar and Gill outline the higher rates of conversion from IGT and/or IFG to type 2 diabetes among South Asian population than among white populations in the UK [17].

## Strategies for the prevention of diabetes

Primary prevention refers to the prevention of the onset of disease. It is also appropriate to include primordial prevention (i.e. preventing the uptake of diabetes risk factors) in the primary prevention of type 2 diabetes, as suitable strategies instituted in potential mothers at the preconception stage reduce the risk of malnutrition and its ill-effects [18]. Earlier studies on preventive aspects of type 2 diabetes from developed and developing countries have given hope by showing that type 2 diabetes is a preventable disorder [4, 7, 19]. The possibility of preventing type 2 diabetes using lifestyle changes and insulin sensitisers has been proven [20]. Physical activity, a healthy diet and a stress-free environment are congenial to a healthy life, and need to be cultivated early in life and practiced during the ‘life circle’ [21], which refers to the continuum from conception through pregnancy, infancy and childhood to adult life. Primary prevention of type 2 diabetes is possible by modifying major risk factors, such as obesity and insulin resistance.

As shown in Fig. 1, it is important to consider the socioeconomic aspects and availability of support from the healthcare system when formulating strategies for the implementation of effective prevention programmes [20]. Landmark prevention trials have contributed pioneering observations with regard to the methodology and practicality of type 2 diabetes prevention programmes, as well as giving insight into the mechanisms underlying prevention strategies [22]. These have helped to generate practical guidelines for implementing programmes for the prevention of type 2 diabetes in most LMICs. A recent review of effectiveness studies of lifestyle interventions to prevent type 2 diabetes and gestational diabetes in LMICs found 48 studies published between 2000 and 2022 (none from LICs) from Asia (*n*=41 studies), America (*n*=6 studies) and Africa (*n*=1 study from Egypt) [23]. Studies from India (*n*=15 studies) and China (*n*=12 studies) accounted for 56% of all studies included in the review. Two South African studies were not included in this review: the South African Diabetes Prevention Program (SA-DPP), which is still ongoing [24–26], and the recently completed Lifestyle Africa intervention [27, 28].

### Essential steps for implementation of effective prevention programmes for type 2 diabetes in LMICs

Diabetes prevention programmes implemented in LMICs have generally been adapted from HIC models, in particular the US DPP and the Finnish Diabetes Prevention Study (DPS), with indications that this adaptation always results in a new intervention that should be tested in the new setting before widescale implementation [29, 30]. The implementation of such programmes requires four steps, as shown in Fig. 1 and detailed below.

**Step 1** The first step is the creation of awareness among the community in general, including among the general population and healthcare personnel, such as physicians, nurses and community workers. It is also imperative to create awareness of the need for diabetes prevention programmes, and to sensitise governmental and non-governmental organisations to obtain support and facilities to conduct large-scale community programmes. Awareness creation should target the community and stakeholders, including the government. Public lectures imparted through media have been found to be helpful, as have health fares and educations camps that are held at work sites and residential colonies and organised by medical practitioners and voluntary organisations. Newspapers and mass media can be used effectively to propagate knowledge of the signs and symptoms of type 2 diabetes, the risk factors for this condition and the need to identify the disease at an early state. Initiatives such as the Indian Diabetes Prevention Programmes, the Prevention Awareness Counselling and Evaluation (PACE) diabetes project and the community-based participatory diabetes prevention and management intervention have provided awareness of diet, physical activity and improvement of quality of life through trained trainers and specialised healthcare personnel in urban and rural settings [31]. The success of health education and health communication is influenced by the level of general literacy in the targeted population, and making healthy choices following health education is, to some extent, conditioned by what is offered by the environment. Limited evidence supports a correlation between a built environment and physical activity, cardiovascular risk factors and clinical outcomes [32, 33], with attributes that affect physical activity differing between LMICs and HICs [34].

**Step 2** The second step in implementing an effective prevention programme is the identification of the population to be screened for IGT and/or IFG and other early glycaemic abnormalities. Screening to identify high-risk individuals should adopt a simple method that is acceptable to the public [35, 36]. It should be a modality that can be scaled up to the community level. It must be an economical procedure so that the programme can benefit a large population. The population to be screened, the method of execution of the programme and the mode of delivery of the intervention have to be carefully designed.

Use of risk scores for type 2 diabetes is now available for most ethnic populations. These are non-invasive, affordable and appropriate for large-scale primary screening [37, 38]. Non-invasive risk scores are mostly based on risk factors that are routinely measured in the healthcare setting. By using these risk scores as the primary screening strategy, individuals who are likely to be at risk of IGT and/or IFG, or diabetes can be identified and biological testing using blood samples can be carried out in high-risk groups. This screening procedure using risk scores is cost- and labour-saving, and is acceptable to the population since many individuals are not unnecessarily subjected to screening using blood tests [39]. It is important that consideration is given to population-specific thresholds to define high risk where applicable (e.g. waist circumference and BMI thresholds for defining obesity [40, 41]) when devising or operationalising risk scores.

Opportunistic screening at healthcare centres and hospitals is also useful among people who present for reasons other than diabetes-related complaints. A simple fasting or random blood glucose or HbA_1c_ test can identify people as having diabetes or hyperglycaemia. There exists a general consensus on the threshold of glucose-based tests (fasting glucose, 2 h glucose, random glucose) [42, 43] to confirm the status of diabetes and IFG and/or IGT. There is less agreement on the use of HbA_1c_ to diagnose diabetes due to its variable diagnostic performance in diverse settings. The high prevalence of sickle-cell disease, anaemia and HIV infection across Africa makes HbA_1c_ a less appropriate biomarker of diabetes risk than a glucose-based test [44, 45].

In countries such as those in Africa, risk screening for diabetes could be paired with existing health-screening programmes. For instance, to comply with their commitments to reach the Joint United Nations Programme on HIV/Acquired Immunodeficiency Syndrome (UNAIDS) targets for HIV, screening campaigns in South Africa are expected to reach out to about 10 million people per year [46]. This is an opportunity to implement diabetes risk screening as part of an existing programme, with modest additional investment, and to deliver prevention interventions to those at high risk.

**Step 3** The third step is planning the programme, which should take into consideration the targeted population, practicality of execution and available resources to support the programme. Efficient planning of resource use, training of the study team, methodical data collection and well-defined biological investigations are essential. An effective protocol for supervision and conduct of the programme should be laid down. The availability of a workforce with expertise, and high-quality training are required. Most of the primary prevention studies of type 2 diabetes have used lifestyle modification (LSM) [47], while only a few studies have used pharmacological agents [47]. Lifestyle intervention programmes, with the goal of decreasing excess weight, increasing physical activity, improving diet quality and reducing unhealthy habits (smoking, alcohol intake, stress), have proven efficient at supporting overall behaviour change. The mode of intervention has evolved with time, from individualised one-to-one personal counselling to the use of information technology. Mobile health (mHealth) is found to be useful in facilitating sustained behavioural changes, with a wider reach and at less cost and time relative to resource-intensive approaches such as individualised one-to-one interventions. The Indian SMS study, using mobile phones to modify behaviour among men with IGT, showed a 36% relative risk reduction in the development of diabetes [48]. A large study by Pfammatter et al addressed the utility of an mHealth intervention to improve diabetes risk behaviours using text messaging among one million Indian adult volunteers [49]. The study found text messaging to be effective in improving diabetes-related health behaviours at 6 months’ follow-up compared with usual care.

The high penetration of mobile communication technology in LMICs offers the opportunity of using communication technology to remotely deliver or sustain diabetes prevention interventions [50–52]. Furthermore, mHealth applications can be used to increase the reach of diabetes risk screening (self-screening and self-referral, for instance), and improve linkage to and retention in care for those diagnosed with diabetes during screening. In general, the WHO has recognised the potential of mHealth to transform the face of healthcare delivery worldwide [52].

While a variety of delivery models and implementers exist in real-world scenarios, community-based programmes are preferred in under-resourced LMICs ([53, 54]. Community-based group sessions offer considerable advantages over individual-health facility-based consultations by reducing travel time and cost for participants, and increase participation in the intervention. Community health workers, who are key players in community-delivered strategies, can be used to efficiently deliver diabetes prevention interventions in LMICs [51]. It is crucial that prevention strategies should encompass both social and behavioural factors to influence individual, interpersonal, community and policy decisions.

**Step 4** For the assessment of efficacy of the first three steps in the implementation of an effective prevention programme, it is essential that a systematic outcome evaluation is conducted. The fourth step, therefore, encompasses monitoring the programme, cost analysis and taking effective measures for long-term sustainability of the programme. These are discussed in more detail below.

#### Monitoring the programme

Monitoring diabetes prevention interventions in LMICs should be an integral part of the surveillance of diabetes and non-communicable diseases (NCDs), to track the progression of the disease in people at high risk at population level [55]. For people with IGT and/or IFG, the American Diabetes Association recommends monitoring the development of type 2 diabetes at least on an annual basis, through individual risk assessment [56]. With the advent of the WHO STEPwise approach to NCD risk factor surveillance (STEPS), an increased number of LMICs have been able to collect population-level data on diabetes and other common NCD risk factors [57].

#### Cost-effectiveness

Cost analyses of diabetes prevention in LMICs have been conducted from various perspectives, based on within-trial or simulation modelling studies [58], with some of the first analyses coming from the Indian Diabetes Prevention Programme (IDPP) [59]. In this trial, over the 3 years of the study, the incremental cost-effectiveness ratio (ICER) to prevent one case of diabetes was US$1052 with LSM, US$1095 with metformin, and US$1359 with the combination of both. The number needed to treat (NNT) to prevent one case of diabetes was 6.4, 6.9 and 6.5 in the LSM, metformin and LSM plus metformin groups, respectively [60]. These figures were much lower than those from the US DPP, where the cost per case of diabetes averted was US$15,700 for lifestyle alone and US$31,000 for metformin [61]. In the Diabetes Community Lifestyle Improvement Program (D-CLIP), which was also conducted in India and used a stepped approach to diabetes prevention, the ICER ranged from international (INT)$6705 to INT$22,574 per case of diabetes prevented [62]. In the Kerala DPP, the ICER per case of diabetes prevented was US$95.2 from a healthcare perspective and US$295.1 from a societal perspective, using group-based community-delivered peer-support lifestyle intervention [63]. In the DMagic trial in Bangladesh, the ICERs were INT$316 per case of intermediate hyperglycaemia or type 2 diabetes prevented and INT$6518 per case of type 2 diabetes prevented among individuals with intermediate hyperglycaemia, using community mobilisation led by lay facilitators [64]. Estimated long-term effectiveness of the Da Qing DPS, derived using Markov Monte Carlo models, indicated that the intervention was cost-saving over a 30 year and lifetime horizon [65].

A global systematic review found that among population-based interventions, taxing sugar-sweetened beverages (SSBs) was cost-saving from both health-system and governmental perspectives. However, evaluation of other strategies including fruit and vegetable subsidies, educational programmes (targeting improvements in physical activity and diet) and modifications to the built environment showed inconsistent results [66]. A modelling study from South Africa showed that a 20% SSB tax over a 20 year horizon could substantially reduce diabetes incidence, prevalence and related mortality, translating into over US$860 million of diabetes healthcare costs averted [67].

#### Sustainability over the long term

The spread of diabetes prevention to LMICs has been mostly based on adaptations of the US DPP and Finnish DPS. The outcome of such adaptation efforts is a new intervention that requires rigorous testing in the new setting to demonstrate its effectiveness, prior to any attempt to scale up the intervention [29, 30]. The pooled effect estimates across LMIC studies showed a significant 25% relative risk reduction in incident diabetes, and favourable effects on a range of other CVD outcomes following diabetes prevention programme interventions, confirming that successful adaptation of diabetes prevention programmes in LMICs is possible [23, 68]. The need to adapt the interventions to the country and sub-country level requires the capacity and expertise of individuals within the country in question; these human resources are likely to be lacking in many LMICs. Over the last decade, the Global Alliance for Chronic Diseases (GACD) has undertaken the challenge to develop implementation science capacity and capability in relation to NCDs across LMICs, which will benefit these countries when they consider implementing programmes for diabetes prevention [69]. The success of any prevention programme relies on its acceptability in the community and sustainability, with continued outcome benefits. It is essential, therefore, to gain social support via the involvement of key stakeholders, both at governmental and non-governmental levels.

## Challenges in population approaches to type 2 diabetes prevention in LMICs

Translation of the findings from a research setting to real-world situations poses major hurdles. There are cultural, societal and economic constraints, besides sustaining the behavioural changes over a lifetime, which needs support from family and society at large. In LMICs in particular, insufficient infrastructure and healthcare capacity, and lack of resources make quality improvement programmes challenging (see Textbox: ‘Challenges and barriers in implementing prevention programmes in LMICs’).
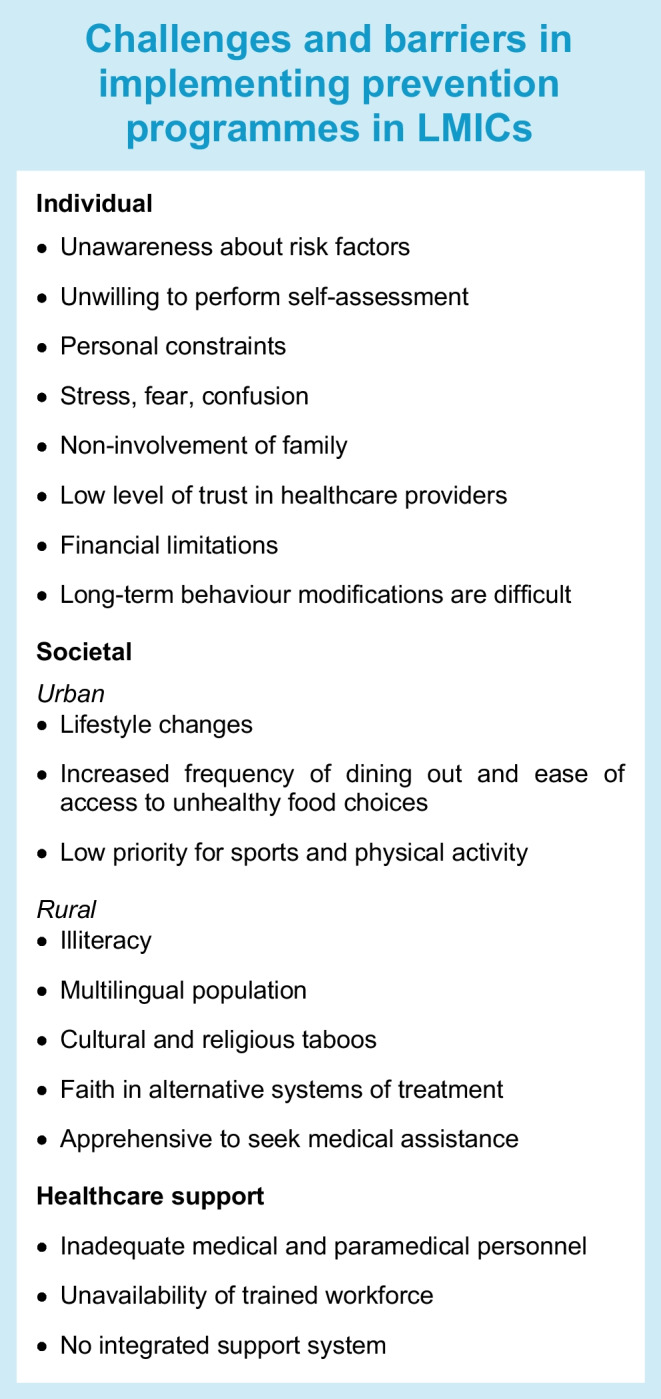


While prevention studies have been conducted in some MICs [70], the spread of these studies remains limited, with none conducted in Africa. The complexity of diabetes pathophysiology and natural history, the variable distribution and impact of the determinants of diabetes and other factors support the need for a widespread demonstration of diabetes prevention effectiveness in LMICs [71].

A review of the implementation of NCD prevention policies across five African countries (Cameroon, Kenya, Malawi, Nigeria and South Africa) up to 2016 found a huge implementation gap, with only one country having evidence on the implementation of some form of policy on physical activity (promotion of physical activity) and diet (public awareness campaigns through mass media) [72]. An analysis of the implementation of 18 NCD policies related to the WHO’s ‘best buys’ interventions (i.e. interventions targeting the main drivers of NCDs) [73] across 151 countries found that, in 2017, the 20 top-performing countries were in Europe and Central Asia (with at least 71% of all policies implemented in these countries), while 17 of the 20 lowest-performing countries were sub-Saharan African countries, with only 5–26% of all policies being implemented in these countries [74].

## Conclusion

In the last two decades, studies from both developed and developing countries have shown the possibility of preventing type 2 diabetes in all ethnicities. However, the questions remain whether prevention programmes are sustainable over the long term and whether they can be scaled up to national levels. In economically weaker countries, management of acute communicable diseases take the centre stage. Therefore, the process of implementing national efforts for the prevention of NCDs, such as diabetes, is seldom given priority. In LMICs, insufficient infrastructure and healthcare capacity, and lack of funding make community-level prevention programmes an insurmountable task, at least in the near future. Taking the next leap, medical personnel and administrators can work together in building an effective workforce to set up registries for risk stratification, disease monitoring and access to quality medical care. Together with stakeholders (healthcare providers, academics and private partnerships), governments should promote diabetes prevention through health literacy, early screening and access to medical support without disparities in the community as whole.

### Supplementary Information

Below is the link to the electronic supplementary material.

Supplementary file1 (PPTX 201 KB)

## Abbreviations

DPP: Diabetes Prevention Program
DPS: Diabetes Prevention Study
GBD: Global burden of disease
HIC: High-income country
ICER: Incremental cost-effectiveness ratio
IFG: Impaired fasting glucose
IGT: Impaired glucose tolerance
INT: International (currency)
LIC: Low-income country
LMIC: Low- and middle-income country
LSM: Lifestyle modification
mHealth: Mobile health
MIC: Middle-income country
NCD: Non-communicable disease
NCD-RisC: Non-Communicable Diseases Risk Factors Collaboration
SSB: Sugar-sweetened beverage

## References

[1] International Diabetes Federation (2021). IDF diabetes atlas.

[2] NCD Risk Factor Collaboration (2016). Worldwide trends in diabetes since 1980: a pooled analysis of 751 population-based studies with 4.4 million participants. Lancet.

[3] Global Burden of Disease 2021 Diabetes Collaborators (2023). Global, regional, and national burden of diabetes from 1990 to 2021, with projections of prevalence to 2050: a systematic analysis for the Global Burden of Disease Study 2021. Lancet.

[4] White M (2016). Population approaches to prevention of type 2 diabetes. PLoS Med.

[5] Hills AP, Misra A, Gill JMR (2018). Public health and health systems: implications for the prevention and management of type 2 diabetes in South Asia. Lancet Diabetes Endocrinol.

[6] Flood D, Seiglie JA, Dunn M (2021). The state of diabetes treatment coverage in 55 low-income and middle-income countries: a cross-sectional study of nationally representative, individual-level data in 680 102 adults. Lancet Healthy Longev.

[7] Chan JCN, Lim LL, Wareham NJ (2021). The Lancet Commission on diabetes: using data to transform diabetes care and patient lives. Lancet.

[8] Wareham NJ, Herman WH (2016). The clinical and public health challenges of diabetes prevention: a search for sustainable solutions. PLoS Med.

[9] Wareham NJ (2015). Mind the gap: efficacy versus effectiveness of lifestyle interventions to prevent diabetes. Lancet Diabetes Endocrinol.

[10] Hu FB (2011). Globalization of diabetes: the role of diet, lifestyle, and genes. Diabetes Care.

[11] Nanditha A, Susairaj P, Raghavan A (2021). Secular trends in cardiovascular risk factors among urban and rural populations in Tamil Nadu, India - an ancillary analysis of the STRiDE-I study. Diabetes Res Clin Pract.

[12] Anjana RM, Deepa M, Pradeepa R (2017). Prevalence of diabetes and prediabetes in 15 states of India: results from the ICMR-INDIAB population-based cross-sectional study. Lancet Diabetes Endocrinol.

[13] Ramachandran A, Snehalatha C, Yamuna A, Murugesan N, Narayan KM (2007). Insulin resistance and clustering of cardiometabolic risk factors in urban teenagers in southern India. Diabetes Care.

[14] Gupta R, Misra A, Vikram NK (2009). Younger age of escalation of cardiovascular risk factors in Asian Indian subjects. BMC Cardiovasc Disord.

[15] Whincup PH, Gilg JA, Papacosta O (2002). Early evidence of ethnic differences in cardiovascular risk: cross sectional comparison of British South Asian and white children. BMJ.

[16] Hasson BR, Apovian C, Istfan N (2015). Racial/ethnic differences in insulin resistance and beta cell function: relationship to racial disparities in type 2 diabetes among African Americans versus Caucasians. Curr Obes Rep.

[17] Sattar N, Gill JM (2015). Type 2 diabetes in migrant south Asians: mechanisms, mitigation, and management. Lancet Diabetes Endocrinol.

[18] Yajnik CS, Deshmukh US (2008). Maternal nutrition, intrauterine programming and consequential risks in the offspring. Rev Endocr Metab Disord.

[19] Misra A (2018). Prevention of diabetes: countless opportunities and clear challenges. Am J Lifestyle Med.

[20] Ramachandran A, Snehalatha C (2011). Diabetes prevention programs. Med Clin North Am.

[21] Wijesuriya M, Williams R, Yajnik C (2010). The Kathmandu Declaration: "Life Circle" approach to prevention and care of diabetes mellitus. Diabetes Res Clin Pract.

[22] Ramachandran A, Snehalatha C, Shetty SA, Nanditha A, Bergman M (2014). Primary prevention trials in type 2 diabetes. Global health perspectives in prediabetes and diabetes prevention.

[23] Sagastume D, Siero I, Mertens E, Cottam J, Colizzi C, Penalvo JL (2022). The effectiveness of lifestyle interventions on type 2 diabetes and gestational diabetes incidence and cardiometabolic outcomes: a systematic review and meta-analysis of evidence from low- and middle-income countries. EClinicalMedicine.

[24] Hill J, Lavigne Delville C, Auorousseau AM (2020). Development of a tool to increase physical activity among people at risk for diabetes in low-resourced communities in Cape Town. Int J Environ Res Public Health.

[25] Hill J, Faber M, Peer N, George C, Oldenburg B, Kengne AP (2023). Adapting and developing a diabetes prevention intervention programme for South Africa: curriculum and tools. Int J Environ Res Public Health.

[26] Hill J, Yako Y, George C, Musarurwa H, Jordaan E, Kengne AP (2023). A study protocol for a cluster randomized controlled trial to test the applicability of the South African diabetes prevention program in the Eastern Cape Province of South Africa. BMC Public Health.

[27] Catley D, Puoane T, Tsolekile L (2019). Adapting the Diabetes Prevention Program for low and middle-income countries: protocol for a cluster randomised trial to evaluate 'Lifestyle Africa'. BMJ Open.

[28] Catley D, Puoane T, Tsolekile L et al (2022) Evaluation of an adapted version of the Diabetes Prevention Program for low- and middle-income countries: a cluster randomized trial to evaluate "Lifestyle Africa" in South Africa. PLoS Med 19(4):e1003964. 10.1371/journal.pmed.1003964 10.1371/journal.pmed.1003964 PMC9053793 35427357

[29] Oldenburg B, Absetz P, Dunbar JA, Reddy P, O'Neil A (2011). The spread and uptake of diabetes prevention programs around the world: a case study from Finland and Australia. Transl Behav Med.

[30] Weber MB, Hassan S, Quarells R, Shah M (2021). Prevention of type 2 diabetes. Endocrinol Metab Clin North Am.

[31] Ranjani H, Weber MB, Narayan KM, Mohan V (2014) Real life diabetes prevention initiative in India. In: Bergman M (ed) Global health perspectives in prediabetes and diabetes prevention. World Scientific Publication, Singapore, pp 281–315. 10.1142/9789814603324_0013

[32] Elshahat S, O'Rorke M, Adlakha D (2020). Built environment correlates of physical activity in low- and middle-income countries: a systematic review. PLoS One.

[33] Malambo P, Kengne AP, De Villiers A, Lambert EV, Puoane T (2016). Built environment, selected risk factors and major cardiovascular disease outcomes: a systematic review. PLoS One.

[34] Cleland C, Reis RS, Ferreira Hino AA (2019). Built environment correlates of physical activity and sedentary behaviour in older adults: a comparative review between high and low-middle income countries. Health Place.

[35] Duan D, Kengne AP, Echouffo-Tcheugui JB (2021). Screening for diabetes and prediabetes. Endocrinol Metab Clin North Am.

[36] Kengne AP, Masconi K, Mbanya VN, Lekoubou A, Echouffo-Tcheugui JB, Matsha TE (2014). Risk predictive modelling for diabetes and cardiovascular disease. Crit Rev Clin Lab Sci.

[37] Ramachandran A, Snehalatha C, Vijay V, Wareham NJ, Colagiuri S (2005). Derivation and validation of diabetes risk score for urban Asian Indians. Diabetes Res Clin Pract.

[38] Saaristo T, Peltonen M, Lindström J (2005). Cross-sectional evaluation of the Finnish Diabetes Risk Score: a tool to identify undetected type 2 diabetes, abnormal glucose tolerance and metabolic syndrome. Diab Vasc Dis Res.

[39] Priscilla S, Nanditha A, Simon M (2015). A pragmatic and scalable strategy using mobile technology to promote sustained lifestyle changes to prevent type 2 diabetes in India-outcome of screening. Diabetes Res Clin Pract.

[40] Alberti KG, Eckel RH, Grundy SM (2009). Harmonizing the metabolic syndrome: a joint interim statement of the International Diabetes Federation Task Force on Epidemiology and Prevention; National Heart, Lung, and Blood Institute; American Heart Association; World Heart Federation; International Atherosclerosis Society; and International Association for the Study of Obesity. Circulation.

[41] Alberti KG, Zimmet P, Shaw J (2007). International Diabetes Federation: a consensus on type 2 diabetes prevention. Diabet Med.

[42] Alberti KG, Zimmet PZ (1998) Definition, diagnosis and classification of diabetes mellitus and its complications. Part 1: diagnosis and classification of diabetes mellitus provisional report of a WHO consultation. Diabet Med 15(7):539–553. 10.1002/(SICI)1096-9136(199807)15:7%3c539::AID-DIA668%3e3.0.CO;2-S 10.1002/(SICI)1096-9136(199807)15:7<539::AID-DIA668>3.0.CO;2-S 9686693

[43] World Health Organization, International Diabetes Federation (2006). Definition and diagnosis of diabetes and intermediate hyperglycemia: report of a WHO/IDF consultation.

[44] Gordon DK, Hussain M, Kumar P, Khan S, Khan S (2020). The sickle effect: the silent titan affecting glycated hemoglobin reliability. Cureus.

[45] Nguyen KA, Peer N, de Villiers A (2019). Glycated haemoglobin threshold for dysglycaemia screening, and application to metabolic syndrome diagnosis in HIV-infected Africans. PLoS One.

[46] The South African National AIDS Council (2022) South Africa's National Strategic Plan for HIV, TB and STIs 2017–2022. Available from: https://sanac.org.za//wp-content/uploads/2017/06/NSP_FullDocument_FINAL.pdf. Accessed 3 July 2023

[47] Haw JS, Galaviz KI, Straus AN (2017). Long-term sustainability of diabetes prevention approaches: a systematic review and meta-analysis of randomized clinical trials. JAMA Intern Med.

[48] Ramachandran A, Snehalatha C, Ram J (2013). Effectiveness of mobile phone messaging in prevention of type 2 diabetes by lifestyle modification in men in India: a prospective, parallel-group, randomised controlled trial. Lancet.

[49] Pfammatter A, Spring B, Saligram N (2016). mHealth intervention to improve diabetes risk behaviors in India: a prospective, parallel group cohort study. J Med Internet Res.

[50] MacPherson MM, Merry KJ, Locke SR, Jung ME (2022). mHealth prompts within diabetes prevention programs: a scoping review. Mhealth.

[51] Hill J, Peer N, Oldenburg B, Kengne AP (2017). Roles, responsibilities and characteristics of lay community health workers involved in diabetes prevention programmes: a systematic review. PLoS One.

[52] WHO Global Observatory for eHealth, Kay M, Santos J, Takane M (2011) mHealth: new horizons for health through mobile technologies - second global survey on eHealth. Available from: https://iris.who.int/bitstream/handle/10665/44607/9789241564250_eng.pdf?sequence=1. Accessed 3 November 2023

[53] Aziz Z, Absetz P, Oldroyd J, Pronk NP, Oldenburg B (2015). A systematic review of real-world diabetes prevention programs: learnings from the last 15 years. Implement Sci.

[54] Babagoli MA, Nieto-Martinez R, Gonzalez-Rivas JP, Sivaramakrishnan K, Mechanick JI (2021). Roles for community health workers in diabetes prevention and management in low- and middle-income countries. Cad Saude Publica.

[55] Echouffo-Tcheugui JB, Yaya S, Joshi R, Narayan KMV, Kengne AP (2018). Population surveillance of cardiovascular diseases in low-income to middle-income countries should leverage existing international collaborations. BMJ Glob Health.

[56] American Diabetes Association Professional Practice Committee (2022). 3. Prevention or delay of type 2 diabetes and associated comorbidities: standards of medical care in diabetes 2022. Diabetes Care.

[57] World Health Organization (2023) Noncommunicable disease surveillance, monitoring and reporting: STEPwise approach to NCD risk factor surveillance (STEPS). Available from: https://www.who.int/teams/noncommunicable-diseases/surveillance/systems-tools/steps. Accessed 18 December 2023

[58] Dahal PK, Rawal LB, Mahumud RA, Paudel G, Sugishita T, Vandelanotte C (2022). Economic evaluation of health behavior interventions to prevent and manage type 2 diabetes mellitus in Asia: a systematic review of randomized controlled trials. Int J Environ Res Public Health.

[59] Ramachandran A, Snehalatha C, Mary S (2006). The Indian Diabetes Prevention Programme shows that lifestyle modification and metformin prevent type 2 diabetes in Asian Indian subjects with impaired glucose tolerance (IDPP-1). Diabetologia.

[60] Ramachandran A, Snehalatha C, Yamuna A, Mary S, Ping Z (2007). Cost-effectiveness of the interventions in the primary prevention of diabetes among Asian Indians: within-trial results of the Indian Diabetes Prevention Programme (IDPP). Diabetes Care.

[61] Diabetes Prevention Program Research Group (2003). Within-trial cost-effectiveness of lifestyle intervention or metformin for the primary prevention of type 2 diabetes. Diabetes Care.

[62] Islek D, Weber MB, Ranjit Mohan A (2020). Cost-effectiveness of a stepwise approach vs standard care for diabetes prevention in India. JAMA Netw Open.

[63] Sathish T, Oldenburg B, Thankappan KR (2020). Cost-effectiveness of a lifestyle intervention in high-risk individuals for diabetes in a low- and middle-income setting: trial-based analysis of the Kerala Diabetes Prevention Program. BMC Med.

[64] Fottrell E, Ahmed N, Morrison J (2019). Community groups or mobile phone messaging to prevent and control type 2 diabetes and intermediate hyperglycaemia in Bangladesh (DMagic): a cluster-randomised controlled trial. Lancet Diabetes Endocrinol.

[65] Hu W, Xu W, Si L (2020). Cost-effectiveness of the Da Qing diabetes prevention program: a modelling study. PLoS One.

[66] Zhou X, Siegel KR, Ng BP (2020). Cost-effectiveness of diabetes prevention interventions targeting high-risk individuals and whole populations: a systematic review. Diabetes Care.

[67] Manyema M, Veerman JL, Chola L, Tugendhaft A, Labadarios D, Hofman K (2015). Decreasing the burden of type 2 diabetes in South Africa: the impact of taxing sugar-sweetened beverages. PLoS One.

[68] Tuomilehto J, Uusitupa M, Gregg EW, Lindstrom J (2023). Type 2 diabetes prevention programs-from proof-of-concept trials to national intervention and beyond. J Clin Med.

[69] Global Alliance for Chronic Diseases (2023) A global alliance of health research funders. Available from: http://www.gacd.org/about. Accessed 2 July 2023

[70] Karachaliou F, Simatos G, Simatou A (2020). The challenges in the development of diabetes prevention and care models in low-income settings. Front Endocrinol.

[71] Kengne AP, Echouffo-Tcheugui JB, Sobngwi E, Mbanya JC (2013). New insights on diabetes mellitus and obesity in Africa-part 1: prevalence, pathogenesis and comorbidities. Heart.

[72] Juma PA, Mapa-Tassou C, Mohamed SF (2018). Multi-sectoral action in non-communicable disease prevention policy development in five African countries. BMC Public Health.

[73] World Health Organization (2017) Tackling NCDs: 'best buys' and other recommended interventions for the prevention and control of noncommunicable diseases. Available from: https://apps.who.int/iris/handle/10665/259232. Accessed 29 June 2023

[74] Allen LN, Nicholson BD, Yeung BYT, Goiana-da-Silva F (2020). Implementation of non-communicable disease policies: a geopolitical analysis of 151 countries. Lancet Glob Health.

