# Supplementary material for: Feasibility of prevention of type 2 diabetes in low- and middle-income countries
Source: Diabetologia. 2024 Feb 15;67(5):763–72. doi: 10.1007/s00125-023-06085-1 (PMC10954968; doi:10.1007/s00125-023-06085-1)
Supplement: Supplementary file 1 — Supplementary file1 (PPTX 201 KB) [file 125_2023_6085_MOESM1_ESM.pptx]

## Slide 1
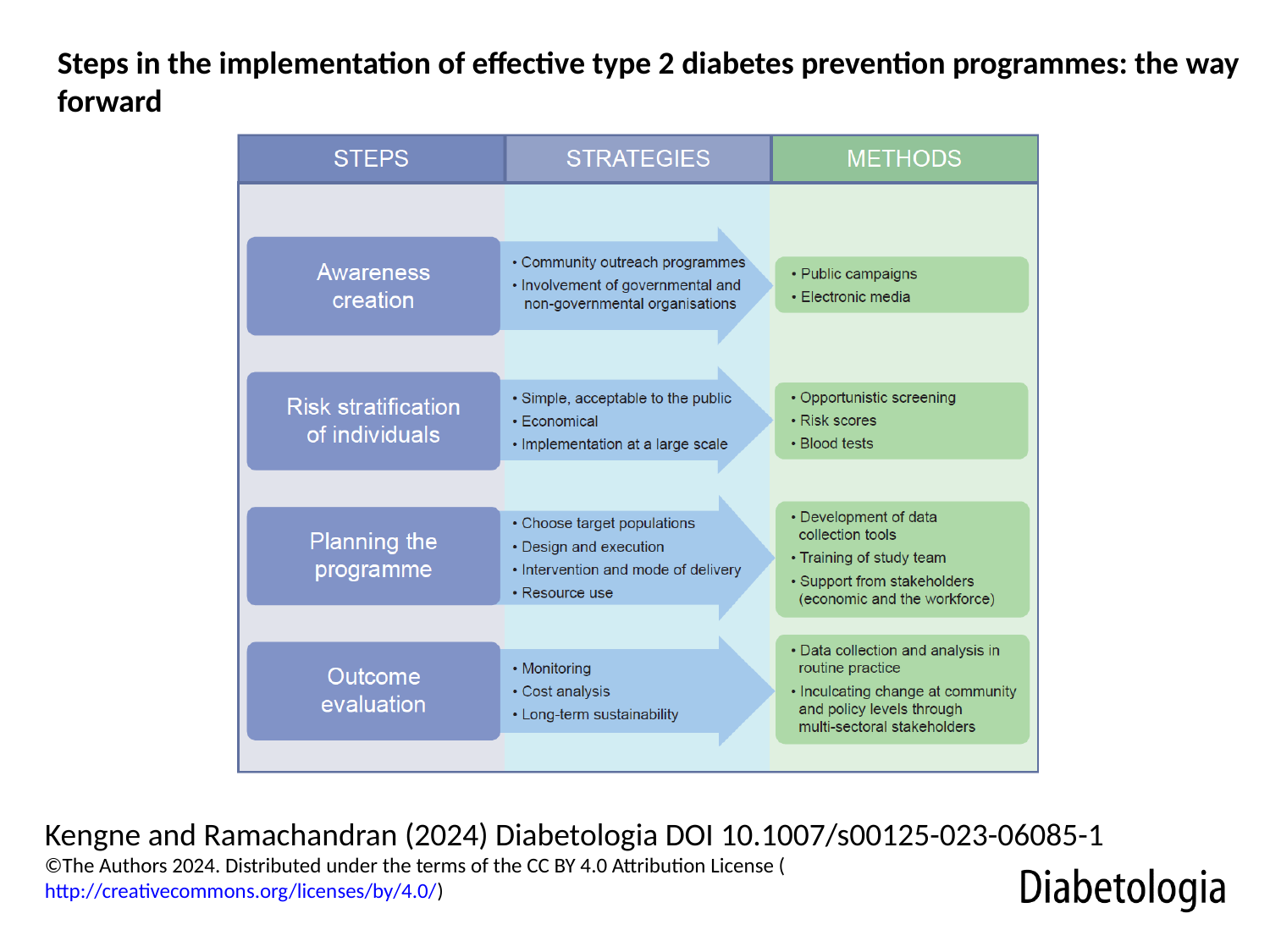

Steps in the implementation of effective type 2 diabetes prevention programmes: the way forward
Kengne and Ramachandran (2024) Diabetologia DOI 10.1007/s00125-023-06085-1
©The Authors 2024. Distributed under the terms of the CC BY 4.0 Attribution License (http://creativecommons.org/licenses/by/4.0/)
